# Noninvasive Hemodynamic Assessment by a Sensor Patch: The PATCHWRK (PortAble TeCHnology for Wireless caRdiomyopathy tracKing) Study

**DOI:** 10.1016/j.jacadv.2025.101753

**Published:** 2025-04-28

**Authors:** Zak Loring, Nicholas B. Bolus, F. Brennan Torstrick, Danielle Wilson, Melissa Lefevre, Brett D. Atwater, Anita Kelsey, Jonathan P. Piccini

**Affiliations:** a Division of Cardiology, Department of Medicine, Duke University, Durham, North Carolina, USA; b Duke Clinical Research Institute, Durham, North Carolina, USA; c Huxley Medical Inc, Atlanta, Georgia, USA; d Inova Schar Heart and Vascular, Falls Church, Virginia, USA

**Keywords:** diastolic function, echocardiography, left bundle branch block, patch sensor

## Abstract

**Background:**

Echocardiographic timing intervals provide prognostic information in patients with preclinical cardiac dysfunction. Reduced diastolic filling time (DFT) identifies left bundle branch block patients at risk for cardiomyopathy. The need for specialized equipment limits the utility of echocardiography (echo) for longitudinal assessment.

**Objectives:**

The purpose of this study was to evaluate a multimodal sensor patch’s (SANSA) assessment of DFT, pre-ejection period (PEP), and left ventricular ejection time (LVET).

**Methods:**

Fifty patients undergoing echo were prospectively enrolled and had simultaneous SANSA patch recording and echo. Timing intervals were analyzed using continuous wave, pulsed wave, and tissue Doppler imaging. SANSA electrocardiogram, seismocardiogram, and phonocardiogram data were independently analyzed to identify valve openings/closures for DFT, PEP, and LVET estimation. Agreement between echo and SANSA estimates was assessed using intraclass correlation coefficients (ICC) and compared with agreement between echo views.

**Results:**

Forty-six of the 50 patients (92%) had analyzable data. The mean ejection fraction was 53% ± 8%; 13 patients (26%) had left bundle branch block. Echo-estimated mean DFT, PEP, and LVET were 416 ± 139 milliseconds (ms), 108 ± 32 ms, and 300 ± 36 ms, respectively. SANSA-estimated DFT, PEP, and LVET were 431 ± 135 ms, 91 ± 35 ms, and 285 ± 43 ms, respectively. The ICC for SANSA vs echo was 0.92 for DFT, 0.74 for PEP, and 0.76 for LVET. The ICC for tissue Doppler imaging vs pulsed wave estimates within the same patients was 0.93 for DFT, 0.83 for PEP, and 0.69 for LVET.

**Conclusions:**

SANSA patch monitoring accurately measures key cardiac timing intervals to within the variability observed between echo views. As these intervals have prognostic value, SANSA-based longitudinal monitoring may facilitate early cardiomyopathy detection.

## Highlights

• Ambulatory assessment of cardiac timing intervals can provide valuable prognostic information in preclinical cardiac dysfunction.
• The multimodal SANSA patch estimates cardiac timing intervals within the error observed between echocardiographic views.
• Longitudinal monitoring of these intervals may facilitate early recognition and intervention in high-risk patients.

Preclinical heart failure (HF) is an increasingly prevalent condition that frequently progresses to structural heart disease prior to the development of symptoms.^1^^,^^2^ Patients with left bundle branch block (LBBB) have a nearly 4-fold increased risk of developing left ventricular (LV) systolic dysfunction compared to patients with normal QRS duration.^3^ This dysfunction develops over a median time of 4 years, but can range between 2 and 7 years. Guideline-directed medical therapies are less effective at reversing LV systolic dysfunction in the presence of LBBB;^4^^,^^5^ however, cardiac resynchronization therapy (CRT) can improve LV function, HF symptoms, and survival among patients with HF symptoms and a reduced ejection fraction.^6^^,^^7^ Earlier employment of CRT improves the probability of LV reverse remodeling.^5^ Recent work has shown that abnormalities in electrocardiographic parameters such as the lead one ratio and echocardiographic diastolic filling time (DFT) can identify patients with LBBB who are at increased risk of developing clinical HF and LV systolic dysfunction, potentially facilitating upstream intervention.^8^^,^^9^ However, 12-lead electrocardiography and echocardiography (echo) require in-person clinical assessment as well as specialized equipment and expertise, limiting its utility as a tool for frequent longitudinal assessments.

A wireless monitoring patch (SANSA, Huxley Medical Inc) that combines multiple sensors capable of capturing seismocardiogram (SCG), electrocardiogram (ECG), photoplethsymogram (PPG), and phonocardiogram (PCG) information has been developed to monitor acute hemodynamic changes during sleep apnea.^10^^,^^11^ The assessment provided by this multimodal patch allows for noninvasive identification of key fiducial points in the cardiac cycle including valve openings/closures with simultaneous ECG. This provides an opportunity to both assess and longitudinally track hemodynamic timing intervals such as DFT, pre-ejection period (PEP), and left ventricular ejection time (LVET) which have been shown to be prognostic in preclinical cardiac dysfunction.^12^ In this study, we evaluated the ability of this noninvasive patch to estimate these timing intervals and compared its performance to the gold standard of echocardiogram performed simultaneously with the patch recording.

## Methods

### Patient population

Patients referred for clinically indicated echo at Duke University Medical Center who met inclusion and exclusion criteria were enrolled at the time of scheduling their echoes. The inclusion criteria required patients to be 18 years of age or older and scheduled for an echocardiogram. Patients were excluded if they had a cardiac implantable electronic device and were paced during the echocardiogram, had complex congenital heart disease (eg, atrioventricular septal defect, ventricular septal defect, aortic coarctation, hypoplastic left heart syndrome, pulmonary atresia, tetralogy of Fallot, total/partial pulmonary venous return, tricuspid atresia, transposition of the great arteries, truncus arteriosus), aortic or mitral stenosis/insufficiency (moderate or greater), or a history of skin irritation from prior medical adhesives. Subjects agreeing to participation reviewed a standard consent form approved by the Institutional Review Board at Duke University Medical Center.

### Sansa patch assessment

**Figure 1:**
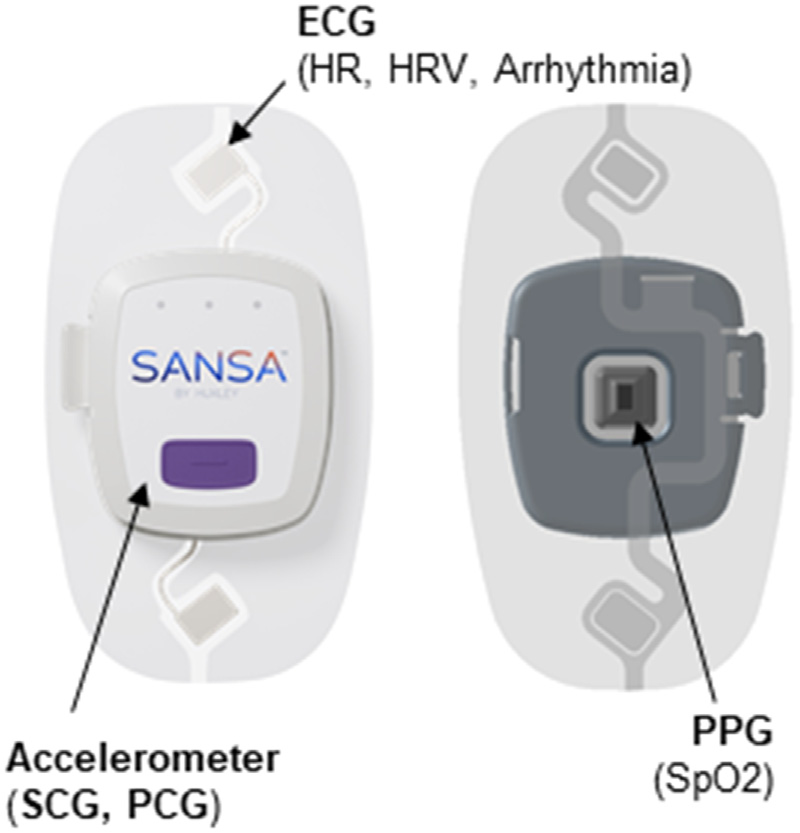
SANSA Patch Multimodal SANSA patch capable of measuring simultaneous electrocardiogram (ECG), seismocardiogram (SCG), and phonocardiogram (PCG). HR = heart rate; HRV = heart rate variability; PPG = photoplethsymogram.

**Figure 2:**
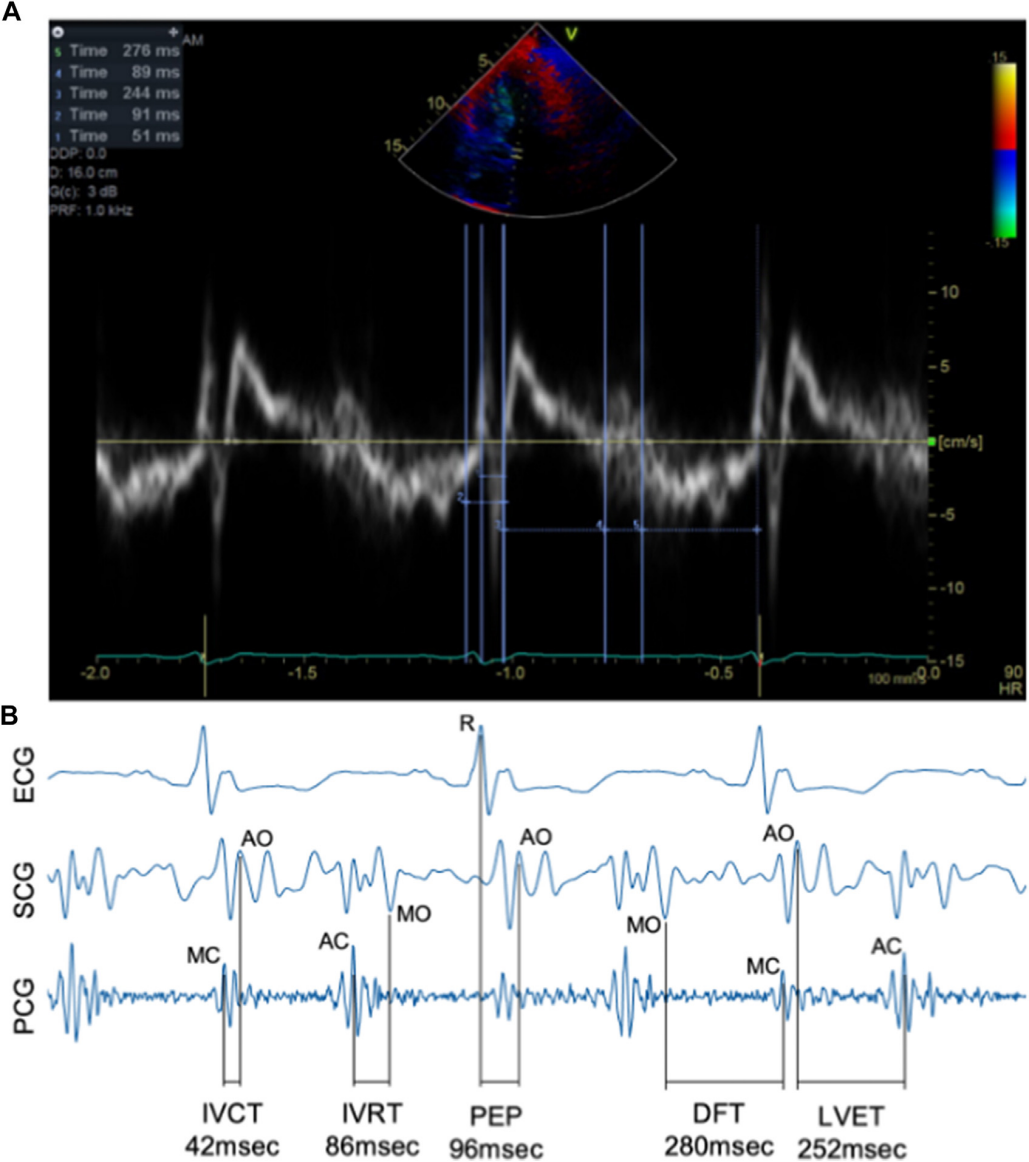
Simultaneous SANSA and Echo Assessment of Pre-Ejection Period, Left Ventricular Ejection Time, and Diastolic Filling Time Tissue Doppler imaging (TDI) of the mitral annulus (A) with simultaneous SANSA patch measurements integrating ECG, SCG, and PCG signals (B) to estimate IVCT (time 1 on TDI image), IVRT (time 4 on TDI image), PEP (time 2 on TDI image), DFT (time 5 on TDI image), and LVET (time 3 on TDI image). AC = aortic closing; AO = aortic opening; DFT = diastolic filling time; ECG = electrocardiogram; IVCT = isovolumetric contraction time; IVRT = isovolumetric relaxation time; LVET = left ventricular ejection time; MC = mitral closing; MO = mitral opening; PCG = phonocardiogram; PEP = pre-ejection period; SCG = seismocardiogram.

At the conclusion of the clinically indicated echocardiographic assessment, patients were prepared for study data collection. Traditional ECG leads were placed as well as a noninvasive blood pressure cuff. The noninvasive wearable sensor patch (SANSA) (Figure 1) was then applied to the chest by trained echo technicians. Patients laid supine for 1 minute prior to echocardiogram acquisition to establish baseline patch measurements. At the completion of the study visit, the patch was removed and its data were downloaded and transferred to a secure electronic database. The patch data were then independently analyzed by trained study personnel blinded to all clinical and echocardiographic data. Key valve actions (aortic valve opening/closure, mitral valve opening/closure) for 5 to 6 cardiac cycles were annotated using a rules-based algorithm that incorporated ECG, SCG, and PCG data. PEP was defined as the interval between QRS onset and aortic valve opening, isovolumetric contraction time (IVCT) was defined as the interval between mitral valve closure and aortic valve opening, LVET was defined as the interval between aortic valve opening and closure, isovolumetric relaxation time (IVRT) was defined as the interval between aortic valve closure and mitral valve opening, and DFT was defined as the interval between mitral valve opening and closure (Figure 2).

### Echocardiographic assessment

Echocardiographic measurements were collected from 3 different views obtained from the apical position. All intervals were assessed using current American Society of Echocardiography guidelines.^13^ At the start of each acquisition, the patch was gently tapped to create an identifiable artifact on both the patch recording and echo ECG to allow for data synchronization and direct comparison of measurements of individual beats. The mitral inflow view with pulsed wave (PW) Doppler was used to document 3 to 5 cardiac cycles and the E-wave onset and DFT were annotated on each beat. The apical five-chamber view with continuous wave (CW) Doppler was then used to acquire 3 to 5 additional cardiac cycles, and the PEP, IVCT, LVET, IVRT, and DFT on each beat. Finally, tissue Doppler imaging (TDI) of the mitral valve septal annulus was performed to acquire 3 to 5 cardiac cycles and the PEP, IVCT, LVET, IVRT, and DFT were measured. The acquired images were then reviewed independently by a physician board-certified in cardiology and echo (A.K.).

### At-home assessment

A subset of patients (N = 5, 10%) participated in the at-home component of the study. This was done to assess the feasibility of at-home use and to compare the quality of signals obtained during in-clinic assessment and ambulatory use. At the conclusion of the in-lab data collection, these patients were trained by clinical personnel on how to place and operate the patch. They were sent home with a SANSA device to wear for 5 minutes of rest while supine. At the end of the at-home period, the device was mailed back to the clinic for data upload and analysis.

### Statistical analysis

Echocardiographic measures of DFT, PEP, and LVET were compared to patch estimates using intraclass correlation coefficients (ICC) and Bland-Altman limits of agreement (LoA). Timing intervals were compared between patch-based estimates, individual echo views (mitral inflow PW, apical five-chamber CW, and mitral annulus TDI), and the average across all echo views. Sensitivity analyses were performed to assess the impact of sex, body mass index (BMI), and presence of LBBB on correlation. Patch estimates were also compared between a patient’s in-lab testing and at-home measurements to explore reliability in an at-home setting. Statistical analyses were performed using R (R Core team 2024).

## Results

Fifty patients who met inclusion and exclusion criteria were enrolled in this study. Baseline demographic and electrocardiographic information is listed in Table 1. The study cohort was 70% female, and 60% self-identified as White/Caucasian. The mean age was 58 ± 13 years, the mean BMI was 31 ± 7 kg/m^2^, and the most common comorbidities were hypertension (62%) and diabetes (32%). A total of 13 patients (26%) had LBBB, 4 patients (8%) had a history of atrial fibrillation, and 13 (26%) had a history of congestive HF.

**Table 1:** Baseline Demographics

Age (y)	58 ± 13
Female	35 (70%)
Self-identified race	
White/Caucasian	30 (60%)
Black	20 (40%)
Other	0 (0%)
Body mass index	31 ± 7
Hypertension	31 (62%)
Diabetes	16 (32%)
Prior myocardial infarction	3 (6%)
Obstructive sleep apnea	16 (8%)
Paroxysmal atrial fibrillation	4 (8%)
Left bundle branch block	13 (26%)
Congestive heart failure	13 (26%)

Values are mean ± SD or n (%).

Echocardiographic parameters are listed in Table 2. The mean heart rate was 74 ± 14 beats/min and mean ejection fraction 52% ± 7%. The absolute values for DFT, PEP, LVET, IVCT, and IVRT differed slightly by echo view. Compared to apical CW mesurements, TDI measurements resulted in longer PEP and IVCT times (11 and 13 ms bias, respectively) and shorter LVET, IVRT, and DFT times (13, 4, and 16 ms bias, respectively). The mean value across all 3 available views was also calculated (Table 2).

**Table 2:** Cardiac Timing Interval Measurements

	DFT	PEP	LVET	IVRT	IVCT
Summary statistics					
Mitral inflow	429 ± 147	-	-	-	-
Apical CW	418 ± 139	102 ± 35	306 ± 38	88 ± 30	65 ± 28
Apical TDI	402 ± 141	113 ± 31	293 ± 40	83 ± 36	78 ± 27
Echo mean	416 ± 139	108 ± 32	300 ± 36	86 ± 30	71 ± 26
Patch	431 ± 135	91 ± 35	285 ± 43	80 ± 26	54 ± 23
Agreement between patch and echo mean					
R	0.92	0.75	0.77	0.47	0.64
Bias	23	−17	−13	−6	−1
LoA	(−81 to 128)	(−65 to 30)	(−67 to 41)	(−63 to 51)	(−58 to 25)
RMSE	58	29	30	30	26
ICC	0.92	0.74	0.76	0.46	0.63
Agreement between TDI and CW					
R	0.93	0.83	0.7	0.62	0.73
Bias	16	−11	13	4	−13
LoA	(−86 to 117)	(−48 to 26)	(−46 to 73)	(−52 to 61)	(−52 to 27)
RMSE	54	22	33	29	24
ICC	0.93	0.83	0.69	0.61	0.73

CW = continuous wave; DFT = diastolic filling time; ICC = intraclass correlation coefficient; IVCT = isovolumetric contraction time; IVRT = isovolumetric relaxation time; LoA = limits of agreement; LVET = left ventricular ejection time; RMSE = root mean squared error; PEP = pre-ejection period; TDI = tissue Doppler imaging.

**Figure 3:**
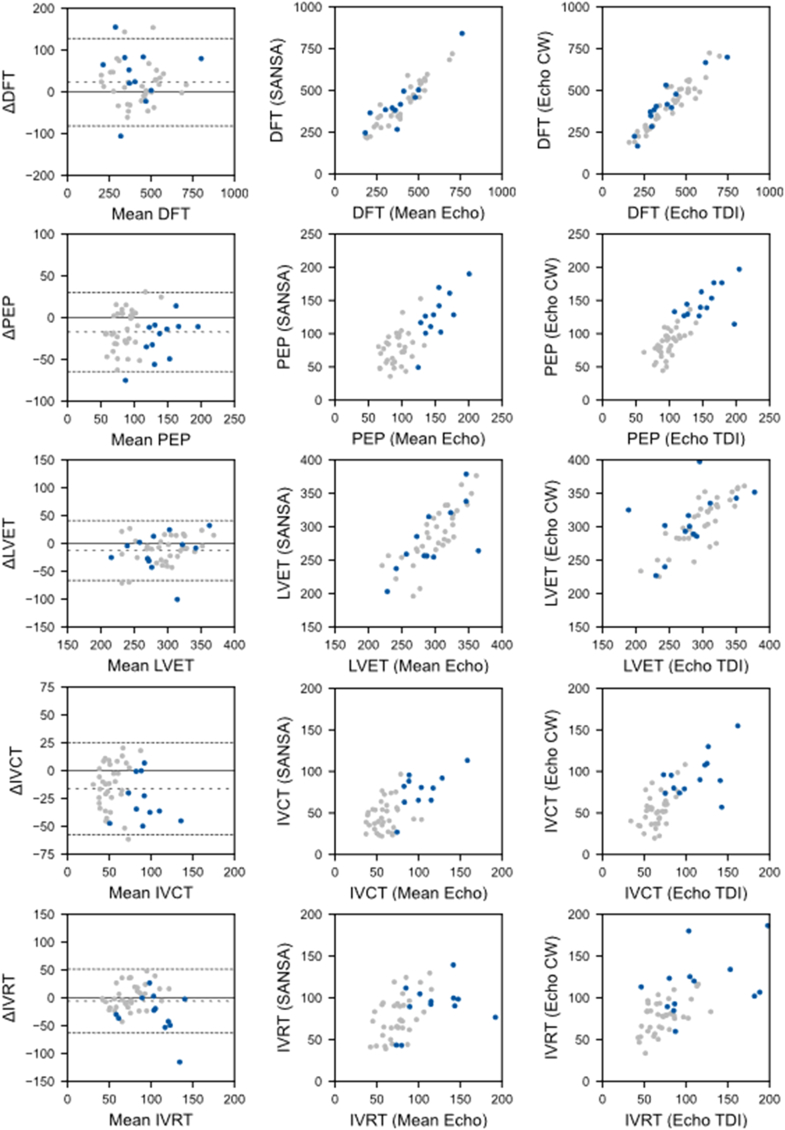
Correlation of SANSA and Echo Timing Intervals Bland-Altman plots and correlation plots comparing SANSA estimates of DFT, PEP, LVET, IVCT, and IVRT to mean echo values as well as between tissue Doppler imaging (TDI) and continuous wave (CW) echo views. Patients with left bundle block are marked in blue. Abbreviations as in Figure 2.

Patch measurements of DFT, PEP, IVCT, LVET, and IVRT were available for 46 out of 50 included patients (92%). ECG data were uninterpretable for 3 patients (6%) precluding timing interval estimation, and one patient (2%) had no detectable mitral closure on the PCG recording (diastolic mitral regurgitation present on echo) precluding estimation of DFT or IVCT. The mean values for the recorded parameters are listed in Table 2. The SANSA-derived mean heart rate was 73 ± 13 beats/min. Compared to the mean echo measurement, patch estimates demonstrated systematically longer DFT (bias 23 ms, LoA −81 to 128 ms) and shorter PEP (bias −17 ms, LoA −65 to 30 ms), LVET (bias −13 ms, LoA −67 to 41 ms), IVRT (bias −6 ms, LoA −63 to 51 ms), and IVCT (bias −16, LoA = −58 to 25 ms). There was a high degree of agreement between DFT estimated by the patch compared to the mean echo estimate (ICC = 0.92; 95% CI: 0.87-0.96; *P* < 0.001) (Figure 3). This level of correlation was similar to that seen between different echo estimation techniques for DFT (apical TDI vs apical CW ICC = 0.93, apical TDI vs mitral inflow PW ICC = 0.91, apical CW vs mitral inflow PW ICC = 0.95). There was strong agreement between the patch and mean echo estimates of PEP (ICC = 0.74 [95% CI: 0.58-0.84], *P* < 0.001) and LVET (ICC = 0.76 [95% CI: 0.61-0.86]), with weaker agreement in estimates of IVCT (ICC = 0.63 [95% CI: 0.43-0.77], *P* < 0.001), and weakest with IVRT (ICC = 0.46 [95% CI: 0.21-0.65], *P* < 0.001) (Figure 3). This level of correlation was also similar to that between different echo techniques (apical TDI vs apical CW: PEP ICC = 0.83 [95% CI: 0.72-0.90], LVET ICC = 0.69 [95% CI: 0.52-0.81], IVCT ICC = 0.73 [95% CI: 0.57-0.84], IVRT ICC = 0.61 [95% CI: 0.40-0.76]).

### Sensitivity analyses

Analyzing men and women independently resulted in a similar level of agreement between patch estimates and mean echo estimates of DFT, with men showing slightly lower correlation in estimates of PEP and LVET, but higher correlation in IVRT estimates (Supplemental Table). Similarly, results were largely consistent across BMI groups with slightly higher correlation estimates of LVET and IVRT among patients with a BMI ≥30. Despite comprising only 26% of the population, patients with LBBB demonstrated similar levels of agreement for all measured parameters as the population at-large, with the exception of IVRT which did not demonstrate a significant correlation with the echo estimates. These trends were similar in subgroup analysis comparing echo apical TDI to apical CW measurements; though among LBBB patients, poor agreement was seen between echo views for all measures except for DFT.

### At-home testing

**Figure 4:**
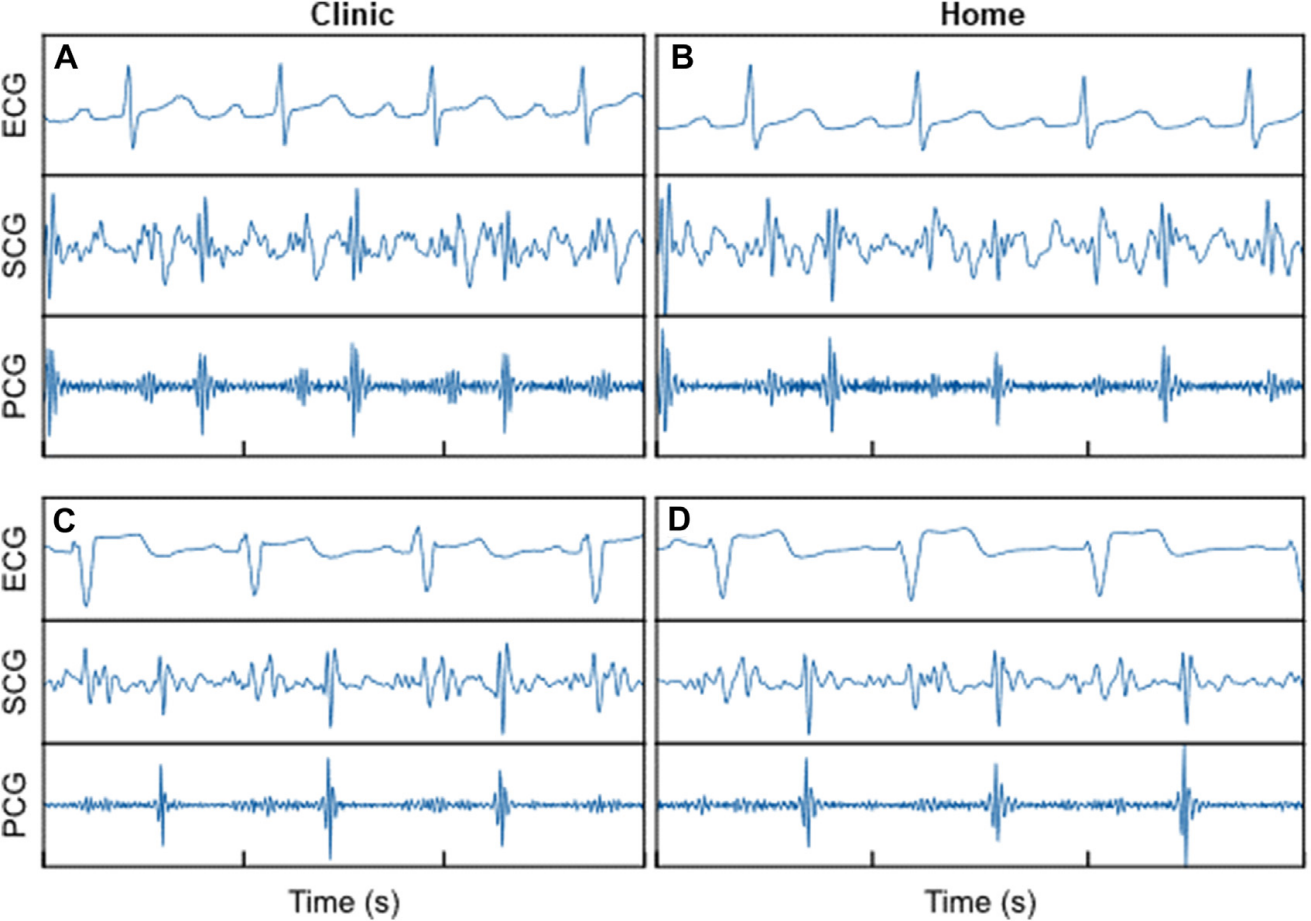
Comparison of In-Clinic and At-Home SANSA Recording In-clinic data (A, C) compared to at-home data (B, D) for 2 subjects (top and bottom rows). Abbreviations as in Figure 2.

Five subjects participated in the at-home substudy. Of those, 4 returned interpretable data for analysis. Side-by-side samples of data acquired in-clinic and data acquired at-home are presented in Figure 4. The at-home recordings had similar if not higher quality signal for the SCG and PCG, with some regions of ECG exhibiting higher noise.

## Discussion

In this study, a noninvasive multimodal sensor patch accurately identified key cardiac timing intervals, demonstrating agreement with echocardiographic estimates comparable to the agreement observed between standard echo views (Central Illustration). Agreement was strongest for DFT, PEP, and LVET, with modest agreement seen in estimates of IVCT and IVRT. At-home testing in a subset of patients demonstrated high-quality assessments of SCG and PCG signals with acceptable ECG signal quality for timing interval estimation, suggesting feasibility of collecting data sufficient quality in unsupervised settings.

**Central Illustration:**
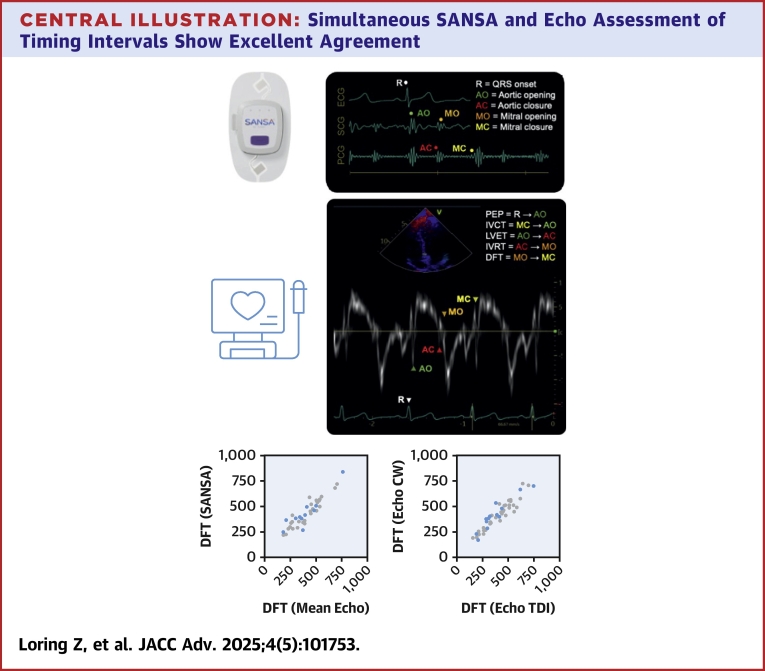
Simultaneous SANSA and Echo Assessment of Timing Intervals Show Excellent Agreement SANSA patch measurements integrating ECG, SCG, and PCG signals (top) identify key cardiac timing intervals with excellent agreement to simultaneously obtained echocardiographic-based measurements (middle). The bottom panel compares agreement between SANSA assessment of DFT and the mean value by echo (left) to the agreement between DFT assessment by continuous wave Doppler (y-axis) and tissue Doppler imaging (x-axis). The agreement between SANSA and echo measurements was similar to the agreement seen between different echo views (bottom). CW = continuous wave; TDI = tissue Doppler imaging; other abbreviations as in Figure 2.

The target population for this study was broad (any patient referred for a clinically indicated, outpatient echocardiogram) in order to capture largely ambulatory patients in whom functional assessment of their cardiac function was deemed appropriate. Enrollment was enriched for patients with a known history of LBBB to ensure at least 25% representation given the prognostic value of DFT in these patients.^8^ Presence of LBBB did not meaningfully impact the agreement seen in any of the studied parameters, though the PEP and IVCT were systematically higher in these patients, as is expected.^14^

The patch estimates demonstrated excellent agreement with echocardiographic estimates when estimating DFT. The patch estimates were slightly higher than the mean echo estimates (bias 23 ms), but 95% of this LoA range fell within the variability observed between individual echo measurements and this error was symmetrically distributed across the range of values. Good agreement was shown between the patch and echo estimates for PEP, LVET, and IVCT, with patch estimates slightly underestimating echo values (bias −17, −12, and −15 ms, respectively) and showing a wider LoA for PEP. IVRT estimates showed weaker agreement for patch vs echo mean and interecho comparisons, which was attributed to the difficulty in identifying points associated with aortic valve closure and mitral valve opening on SANSA and echo images. Overall, IVRT and IVCT were susceptible to the most error given their short duration and occurrence within a single heart sound envelope. IVCT estimation benefits from its position within the higher amplitude S1 heart sound and being terminated by a prominent aortic valve closure. In contrast, IVRT is embedded within the lower amplitude S2 heart sound with less prominent starting and end points.

The patch’s design allows for accurate, simultaneous assessment of multiple modalities. By pairing ECG with SCG and PCG, the timing of key valve actions (opening/closing of the mitral and aortic valves) can be assessed noninvasively. Being able to assess these metrics with a noninvasive patch could allow for remote assessments with minimal resource utilization. Additionally, the promising at-home results suggest that this technology could be used for longitudinal assessment while maintaining high data integrity. The regions of lower quality ECG identified in at-home recordings have been mitigated through subsequent design changes to the early prototype patch used in this study.

Utilization of a noninvasive patch for accurate, longitudinal assessment of key cardiac timing intervals has the potential to improve upstream diagnosis of cardiac dysfunction and possible early intervention to mitigate downstream disease. Large randomized trials have demonstrated that delaying initiation of guideline-directed medical therapy can increase the risk of adverse clinical outcomes such as HF hospitalization or death.^15^^,^^16^ An analysis of longitudinal clinical outcomes comparing patients who were first diagnosed with HF in the outpatient setting compared to those whose diagnosis first came when hospitalized for HF demonstrated a near two-fold increase in hazard of cardiovascular death or HF hospitalization, particularly among patients with preserved ejection fractions.^17^ Implementation of serial outpatient monitoring of patients at risk for developing cardiomyopathy could facilitate early identification of diastolic dysfunction, initiation of appropriate medical therapy, and mitigation of adverse clinical outcomes. Patients with LBBB are at risk for developing cardiomyopathy,^3^ but current guidelines do not recommend CRT until symptoms develop or the ejection fraction drops.^18^^,^^19^ Future studies could leverage this prognostic information to evaluate whether upstream CRT in high-risk patients with LBBB would improve clinical outcomes.

Longitudinal monitoring of patients with known HF could also help inform management decisions. Implantable monitors and algorithms embedded in cardiac implantable electronic devices can use embedded sensors to assess heart sounds, thoracic impedance, heart rate, and/or activity to assess the risk of an impending HF event up to a month prior to its clinical presentation.^20^^,^^21^ While these sensors require surgical implantation of hardware, other devices aim to achieve similar results utilizing noninvasive assessments.^22^ A randomized controlled trial compared clinical outcomes of HF patients with management guided by an acoustic cardiography device evaluating cardiac timing intervals vs usual care and demonstrated a 40% lower hazard of HF hospitalization or death with a device-guided approach.^23^ Device assessment in this study was done during an in-person clinic visit. Ambulatory application of multisensor patch technology has also been able to identify precursors to HF hospitalization with a median 6.5 day lead time.^24^ The present study highlights the utility of multisensor patch technology to impact upstream care in patients with and without known cardiomyopathy while providing correlation to cardiac indices known to be predictive of clinical outcomes.^12^

This study has several important limitations. The target population was relatively broad, including patients with and without either systolic dysfunction and/or LBBB. This heterogeneity may have contributed to some of the variability in the results; however, our prespecified subgroup analyses did not show major group-wise differences in the correlations observed. Patients with cardiac pacing and/or significant congenital or valvular heart disease were excluded and these results cannot be extrapolated to these populations. While analysis of SANSA patch data was fully blinded to echocardiographic data, valve action annotations were performed manually using a rules-based approach.^25^ Current work is focused on automating these annotations to facilitate larger scale applications. Longitudinal changes and/or clinical outcomes were not assessed in the current cohort; thus, the ability for the patch to identify patients at risk for cardiomyopathy is uncertain. While the present study shows that acutely, the SANSA patch demonstrates similar variability in measurement as echo, future work will focus on the longitudinal stability of these measures, the time course of change, and the correlation of these changes with clinical status.

## Conclusions

Noninvasive patch monitoring can accurately identify key cardiac timing intervals including DFT, PEP, LVET, IVCT, and IVRT. The ability to monitor these values noninvasively may allow for earlier intervention and longitudinal ambulatory monitoring of patients for early signs of hemodynamic decline. Upstream identification of cardiac dysfunction may help prevent or reverse the development of systolic dysfunction in patients at risk such as those with LBBB.

Perspectives **COMPETENCY IN MEDICAL KNOWLEDGE:** A noninvasive multisensor patch can accurately estimate key cardiac timing intervals that have prognostic significance.

**TRANSLATIONAL OUTLOOK:** This may allow for longitudinal, ambulatory assessment of cardiac function in patients with risk factors for cardiomyopathy including those with LBBB to facilitate early identification and intervention.

## Funding support and author disclosures

This work was funded by 10.13039/100000002NIH grant 1R43HL158282. Dr Loring has received grant support from 10.13039/100008497Boston Scientific and the Department of Veterans Affairs and has served as a consultant for Huxley Medical Inc and Boston Scientific. Dr Piccini is supported by R01AG074185 from the 10.13039/100000049National Institutes of Aging; has received grants for clinical research from Abbott, the American Heart Association, the Association for the Advancement of Medical Instrumentation, Bayer, Boston Scientific, iRhythm, and Philips; and also has served as a consultant to Abbott, Abbvie, Ablacon, Altathera, ARCA Biopharma, Bayer, Biotronik, Boston Scientific, Bristol Myers Squibb (Myokardia), Element Science, Huxley Medical Inc, Itamar Medical, LivaNova, Medtronic, Milestone, ElectroPhysiology Frontiers, ReCor, Sanofi, Philips, and Up-to-Date. Dr Atwater has received research support from 10.13039/100004374Medtronic and 10.13039/100004319Pfizer and has served as a consultant to Medtronic, Biosense Webster, and Cortex Medical. All other authors have reported that they have no relationships relevant to the contents of this paper to disclose.
