# Supplementary material for: Noninvasive Hemodynamic Assessment by a Sensor Patch: The PATCHWRK (PortAble TeCHnology for Wireless caRdiomyopathy tracKing) Study
Source: JACC Adv. 2025 Apr 28;4(5):101753. doi: 10.1016/j.jacadv.2025.101753 (PMC12242580; doi:10.1016/j.jacadv.2025.101753)
Supplement: Supplemental Table [file mmc1.pdf]

**Supplementary table:** Sensitivity analyses

| Subgroup | Measure | ICC Patch v Echo Mean | ICC TDI vs CW |
|---|---|---|---|
| **Men (N=15)** | DFT | 0.91 [0.76-0.97] | 0.94 [0.84-0.98] |
| | PEP | 0.60 [0.14-0.84] | 0.80 [0.50-0.93] |
| | LVET | 0.53 [0.05-0.81] | 0.76 [0.41-0.91] |
| | IVCT | 0.63 [0.20-0.86] | 0.64 [0.21-0.86] |
| | IVRT | 0.66 [0.24-0.87] | 0.68 [0.28-0.88 |
| **Women (N=45)** | DFT | 0.93 [0.87-0.97] | 0.93 [0.86-0.96] |
| | PEP | 0.79 [0.62-0.89] | 0.83 [0.68-0.91] |
| | LVET | 0.82 [0.68-0.91] | 0.70 [0.48-0.84] |
| | IVCT | 0.67 [0.44-0.82] | 0.75 [0.55-0.86] |
| | IVRT | 0.39 [0.07-0.64] | 0.59 [0.33-0.77] |
| **BMI<30 (N=28)** | DFT | 0.91 [0.83-0.96] | 0.92 [0.84-0.96] |
| | PEP | 0.78 [0.58-0.89] | 0.79 [0.59-0.90] |
| | LVET | 0.70 [0.45-0.85] | 0.67 [0.40-0.83] |
| | IVCT | 0.61 [0.31-0.80] | 0.69 [0.43-0.84] |
| | IVRT | 0.39 [0.03-0.66] | 0.72 [0.48-0.86] |
| **BMI>30 (N=22)** | DFT | 0.93 [0.78-0.97] | 0.94 [0.85-0.97] |
| | PEP | 0.68 [0.37-0.85] | 0.92 [0.81-0.96] |
| | LVET | 0.83 [0.63-0.92] | 0.69 [0.38-0.86] |
| | IVCT | 0.68 [0.37-0.85] | 0.83 [0.63-0.93] |
| | IVRT | 0.61 [0.31-0.83] | 0.40 [-0.02-0.70]* |
| **LBBB (N=13)** | DFT | 0.91 [0.73-0.97] | 0.93 [0.80-0.98] |
| | PEP | 0.67 [0.21-0.88] | 0.51 [-0.03-0.82]* |
| | LVET | 0.69 [0.25-0.89] | 0.51 [-0.03-0.82]* |
| | IVCT | 0.61 [0.11-0.86] | 0.47 [-0.08-0.80]* |
| | IVRT | 0.33 [-0.25-0.73]* | 0.41 [-0.16-0.77]* |

DFT = diastolic filling time; PEP = pre-ejection period; LVET = left ventricular ejection time; IVRT = isovolumetric relaxation time; IVCT = isovolumetric contraction time, BMI = body mass index; LBBB = left bundle branch block. *Not significantly correlated.
